# The Flexible Nature of Unconscious Cognition

**DOI:** 10.1371/journal.pone.0025729

**Published:** 2011-09-28

**Authors:** Martijn E. Wokke, Simon van Gaal, H. Steven Scholte, K. Richard Ridderinkhof, Victor A. F. Lamme

**Affiliations:** 1 Cognitive Neuroscience Group, Department of Psychology, University of Amsterdam, Amsterdam, The Netherlands; 2 Inserm, Cognitive Neuroimaging Unit, Gif-sur-Yvette, France; 3 Commissarìat à l'Energie Atomique, Neurospin Center, Gif-sur-Yvette, France; 4 Department of Psychology, Amsterdam Center for the Study of Adaptive Control in Brain and Behavior (Acacia), University of Amsterdam, Amsterdam, The Netherlands; 5 Cognitive Science Center, University of Amsterdam, Amsterdam, The Netherlands; Northwestern University, United States of America

## Abstract

The cognitive signature of unconscious processes is hotly debated recently. Generally, consciousness is thought to mediate flexible, adaptive and goal-directed behavior, but in the last decade unconscious processing has rapidly gained ground on traditional conscious territory. In this study we demonstrate that the scope and impact of unconscious information on behavior and brain activity can be modulated dynamically on a trial-by-trial basis. Participants performed a Go/No-Go experiment in which an unconscious (masked) stimulus preceding a conscious target could be associated with either a Go or No-Go response. Importantly, the mapping of stimuli onto these actions varied on a trial-by-trial basis, preventing the formation of stable associations and hence the possibility that unconscious stimuli automatically activate these control actions. By eliminating stimulus-response associations established through practice we demonstrate that unconscious information can be processed in a flexible and adaptive manner. In this experiment we show that the same unconscious stimulus can have a substantially different effect on behavior and (prefrontal) brain activity depending on the rapidly changing task context in which it is presented. This work suggests that unconscious information processing shares many sophisticated characteristics (including flexibility and context-specificity) with its conscious counterpart.

## Introduction

For a long time the extent of unconscious information processing has been assumed to be limited in scope and restricted to relatively “low-level” automatic cognitive processes, such as motor preparation. However, in the last decade the boundaries of unconscious cognition have been pushed further and further. Accumulating evidence demonstrates that unconscious information processing can influence behavior or trigger cortical activity previously seen as the domain of conscious cognition. For example, it has been shown that subliminal priming can be modulated by several top-down settings of the cognitive system such as temporal attention [1], [2], [3], spatial attention [1], [4], [5], [6], [7], [8], [9], task-set [10], [11], [12], and strategy or intentions [13], [14], [15], [16]. Further, unconscious information is probably processed all the way up to semantic analysis [17], [18], [19], [20]. Recently unconscious information has been observed to influence even “high-level” cognitive functions, such as task selection [21], inhibitory control [11], [22] and decision-making [23]; and to engender activation of areas in prefrontal cortex (PFC) at high levels of the cognitive and cortical hierarchy.

Although these (and more) studies have revealed that unconscious information processing is relatively sophisticated, critics might still argue that the evidence for (high-level) unconscious cognition is often obtained in situations in which the unconscious stimulus is consistently and frequently paired with task performance on the same conscious stimulus. Then, after (substantial) practice, unconscious stimuli are able to trigger behavioral and neural effects, possibly because of increased stimulus-response (S-R) associations [24]. This interpretation is strengthened by several studies that have demonstrated a lack of transfer from trained conscious stimuli to untrained (novel) unconscious stimuli of the same category [24], [25], [26], [27], suggesting that unconscious influences on behavior might actually be mediated by strong sensory-motor links established through learning. Based on these results, one can argue that unconscious information processing still does not escape the realm of processes labeled as automatic, low-level and bottom-up as opposed to the more flexible nature of conscious processing. However, others have found transfer effects from practiced to unpracticed (novel) items [13], [14], [28], [29], which triggered considerable controversies [see 30 for a review].

Here we test whether unconscious stimuli can trigger cognitive control processes in a goal-directed and flexible fashion or whether this capability is restricted to cases where information is perceived consciously. To do so, we designed a Go/No-Go paradigm in which a target stimulus is preceded by an unconscious prime stimulus. The unconscious prime could be associated with either a Go or a No-Go response, determined on a trial-by-trial basis. Therefore, participants had to update S-R associations dynamically and flexibly on every trial (excluding strong S-R learning, see also [31]). By measuring psychophysics and EEG we show that, even when strong S-R associations cannot be formed through learning, an unconscious No-Go stimulus can still trigger PFC-mediated inhibitory control processes [11], [22], [32], suggesting that unconscious cognition is rather flexible and that it might share several sophisticated properties with its conscious counterpart.

## Results

### Task overview

**Figure 1:**
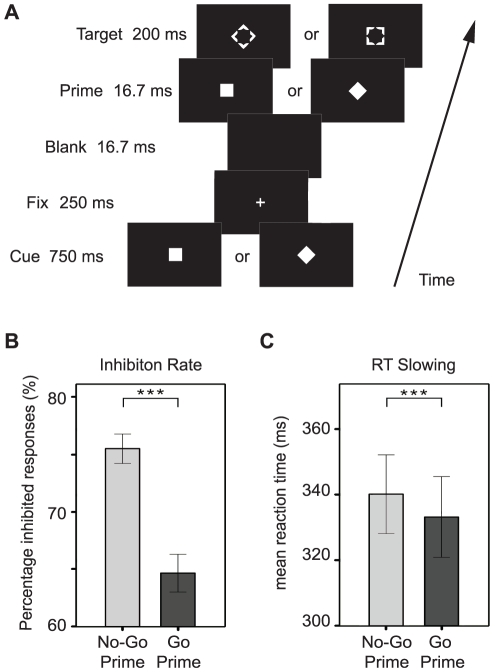
Task design and behavioral results. A) Participants responded to a Go target and attempted to withhold their response on a No-Go target. The target could be a diamond or a square shape depending on the instruction cue that signaled which of both stimuli functioned as the No-Go target in the upcoming trial. Importantly, an unconscious (masked) prime was presented briefly before the (metacontrast) target. Note that prime identity and target identity (being associated with a Go or No-Go response) varied from trial to trial. B) Unconscious No-Go primes when preceding a No-Go target resulted in an increase of 10.8% of the inhibition rate compared to unconscious Go primes. C) Unconscious No-Go primes slowed-down responded Go target trials compared to unconscious Go primes.

Participants (N = 27) were instructed to respond as fast as possible to a Go target by pressing a button with their right index finger and to withhold their response when they perceived a No-Go target. The target could be a diamond or a square shape (see Fig. 1a). Crucially, at the beginning of each trial an instruction cue signaled which of both stimuli (square or diamond) functioned as the No-Go target in the upcoming trial. To study the effect of unconscious information on behavior and brain responses an unconscious prime (square or diamond) was presented briefly before the target. Importantly, the same prime stimulus was associated with a No-Go response on one trial, but with a Go response on the next (depending on the nature of the instruction cue). Therefore, stimulus-response associations changed on a trial-by-trial basis. Importantly, the prime was strongly masked by the meta-contrast target. The 2-choice discrimination task, administered after the main experiment to assess whether primes were indeed not consciously perceived, revealed that 24 out of 27 participants scored at chance-level (chance-level = 50%, binominal test). Although the other three participants who scored slightly above chance-level reported not to have seen the primes (subjectively), the possibility could not be excluded that these participants perceived the primes consciously on some occasions. Therefore, these three participants were excluded from further analyses. Mean discrimination performance across the 24 included participants was 49.3% (SD = 4.3), highlighting that they could not perceive the primes consciously.

### Behavior: unconscious No-Go stimuli trigger response inhibition

Reaction times (RTs) on Go targets (mean RT = 336.6; SD = 59.6) and inhibition rates on No-Go targets (mean inhibition rate = 70.1%, SD = 6.0) were comparable to previous Go/No-Go studies. Intriguingly, although primes were rendered invisible, participants inhibited their responses more often when a No-Go target was preceded by a No-Go prime than when it was preceded by a Go prime (*t*(23) = 6.49, *p*<0.001), indicating that unconscious primes affected inhibitory performance on subsequent No-Go targets (see Fig. 1b). Although prime identity had no influence on the (near perfect) performance scores on Go trials (Go prime preceding Go target: mean percentage correct = 98.4%, SD = 1.7; No-Go prime preceding Go target: mean percentage correct = 98.6%, SD = 2.0; *t*(23) = 1.36, *p* = 0.185), RTs were significantly slower to Go targets preceded by a No-Go prime compared to RTs on Go targets preceded by a Go prime (*t*(23) = 4.09, *p*<0.001), as if participants attempted to inhibit their response but failed to do so entirely (see Fig. 1c). Although not successful as such, the attempt to inhibit may have resulted in a slower buildup of response activation leading to slower responses. Thus, although prime (and target) identity changed on a trial-by-trial basis and therefore strong and stable prime-response associations could not be formed during testing, No-Go primes were still able to trigger response inhibition unconsciously, either by increasing the inhibition rate on conscious No-Go targets, or by slowing down responses to conscious Go targets.

Because stimulus identity changed randomly across trials, on some trials the identity of the No-Go stimulus repeated from one trial to the next, whereas on other trials it changed. Theoretically, it could be that the observed behavioral effects were completely due to rapidly learned S-R associations on “repeat trials” (one-trial learning). To test this alternative hypothesis we re-analyzed the data and divided our dataset into two parts; repeat trials (same No-Go stimulus as on previous trial) and change trials (different No-Go stimulus as on previous trial). If our behavioral effects were caused by fast S-R learning because of repeating the same stimulus across trials one would expect to observe larger behavioral effects for repeat trials compared to change trials. This was not the case. Unconscious RT slowing was present in both repeat trials (*t*(23) = 2.72, *p* = 0.012) and change trials (*t*(23) = 3.58, *p* = 0.002). Further, unconscious inhibition effects were both significant for repeat (*t*(23) = 5.12, *p*<0.001) as well as change trials (*t*(23) = 6.58, *p*<0.001). To test whether unconscious RT slowing or unconscious inhibition effects interacted with trial type (repeat vs. change trials) we performed 2×2 (prime identity×trial type) repeated measures ANOVAs for RTs and inhibition rates separately. No such interactions were observed for unconscious RT slowing (F(1,23) = 0.81, *p* = 0.38) or unconscious inhibition effects (F(1,23) = 2.84, *p* = 0.11); note that, if anything, these effects were larger for change trials. Again, these analyses demonstrate that unconscious information processing is very flexible and does not need (rapid) S-R learning to sort its effects.

### EEG: unconscious No-Go stimuli trigger prefrontal event related potentials

**Figure 2:**
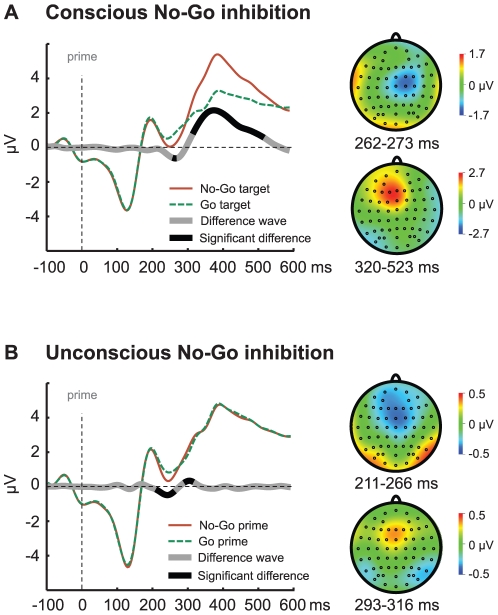
EEG results. A) Conscious No-Go targets elicited a larger N2 and P3 component than conscious Go targets. B) Unconscious No-Go primes elicited a larger N2 and P3 component than unconscious Go primes. The vertical dotted line represents prime presentation. ERPs are computed for a cluster of frontocentral electrodes of interest (Fz, F1, F2, FCz, FC1, FC2, Cz, C1 and C2).

Figure 2a shows prime-locked ERPs for trials containing a No-Go target and trials containing a Go target collapsed across prime identity (No-Go prime, Go prime) as well as the difference wave (No-Go minus Go). Therefore, this comparison shows the brain responses related to the *conscious* activation/implementation of response inhibition while controlling for possible low-level congruency effects at the same time (for details see Methods). Replicating typical ERP findings, we observed a larger frontocentral N2 (peak latency = 270 ms, peak difference = 0.70 µV; significant interval = 262–273 ms, *p*<0.05) and P3 (peak latency = 383 ms; peak difference = 2.33 µV; significant interval = 320–523 ms, *p*<0.05) component for No-Go targets compared to Go targets. Both components peaked at the expected scalp sites and at typical latencies [33], [34], [35], [36], [37], [38].

**Figure 3:**
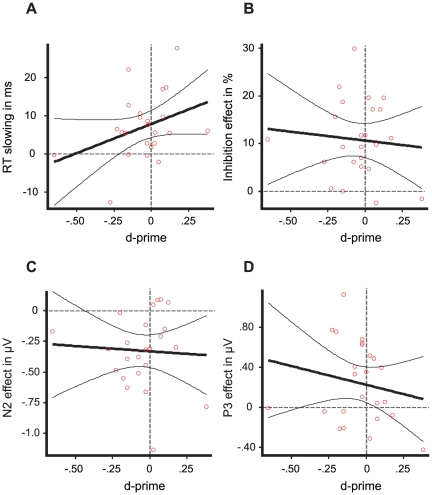
Behavioral and ERP indices of unconsciously triggered inhibition. Linear regression plots showing extrapolated prime-visibility to the point where the discrimination task showed zero sensitivity (d′ = 0). Regression plots are adjoined by their 95% confidence boundaries. Regression of RT slowing (A) and inhibition rate (B) against d′. Regression of the mean (prime identity related) activity differences of the N2 (C) and the P3 (D) component against d′.

To investigate whether masked primes (association varied on a trial-by-trial basis) also triggered a larger N2 and P3 component, we isolated brain activity related to the *unconscious* activation of response inhibition (figure 2b). We compared trials with a No-Go prime with trials with a Go prime (prime-locked, collapsed across target identity and therefore controlling for low-level congruency effects). Crucially, unconscious No-Go primes elicited a larger N2 (peak latency = 242 ms; peak difference = 0.43 µV; significant interval = 211–266 ms, *p*<0.05) and P3 (peak latency = 309 ms; peak difference = 0.28 µV; significant interval = 293–316 ms, *p*<0.05) component than unconscious Go primes. If these ERP effects were caused by fast S-R learning one would expect to observe larger ERP effects for repeat trials compared to change trials. As with behavior, this was not the case. A repeated measures ANOVA revealed that there were no interactions between trial type (repeat vs. change) and prime identity (Go vs.No-Go) showing that unconsciously triggered electrophysiological indices of response inhibition were not modulated by whether the direct previous trial was the same or different (F(1,23) = 0.31, *p* = 0.59). Further, besides the main effect of prime identity (F(1,23) = 40.86, *p*<0.001) showing larger ERP components for No-Go primes compared Go primes, we also observed a main effect of ERP component (F(1,23) = 4.50, *p* = 0.05). This latter effect highlights that the unconscious ERP effects were larger for the N2 than for the P3, nicely confirming previous findings using a stop-signal task including unconscious stop-signals (not aimed at studying the flexibility [39]). Overall, these results show that unconscious stimuli not only activate cognitive control networks in prefrontal cortex, as has been shown before [11], [22], but that they do so in a highly flexible and non-automatic manner. The latencies as well as the scalp distributions were highly comparable with the consciously evoked ERP components, although the strength of both components was considerably smaller [39]. To rule out that any of these effects were caused by accidental prime visibility, discrimination performance in the 2-choice discrimination task (percentage correct) was correlated with ERP (mean difference in significant interval for the N2 and P3) and behavioral indices of unconscious inhibition (RT slowing and inhibition rates). None of these correlations approached significance (all *p*s>0.25). Furthermore, we extrapolated prime-visibility to the point where the discrimination task showed zero sensitivity (d′ = 0) to test whether behavioral and ERP indices of unconsciously triggered inhibition were still observed (figure 3). Regression of RT slowing and inhibition rate against d′ resulted in a significant intercept for RT slowing (intercept = 7.8 ms; *p*<0.001) and inhibition rate (intercept = 10.6%; *p*<0.001). At the point where the discrimination task showed zero sensitivity we also still observed typical ERP indices of inhibition triggered by masked primes. Linear regression of mean activity differences of the N2 and the P3 components against d′ resulted in a significant intercept for the N2 effect (intercept = −0.33 µV; *p*<0.001) and P3 effect (intercept = 0.23 µV; *p* = 0.015) [40], [41].

## Discussion

In this study we explored the flexibility of unconscious information processing in the human brain. To this end, we designed a masked Go/No-Go experiment in which an unconscious prime stimulus preceding a target could either be associated with a Go or No-Go response. Because the identity of the prime was varied on a trial-by-trial basis participants had to update S-R associations dynamically and flexibly on every trial. This experimental set-up allowed us to test whether PFC-mediated cognitive control processes [11], [22], [32] can be triggered in a flexible, non-automatic manner.

Behaviorally, unconscious No-Go primes preceding No-Go targets significantly increased inhibition rates compared to unconscious Go primes. Further, unconscious No-Go primes presented before Go targets slowed-down responses compared to unconscious Go primes. Replicating typical EEG findings, we observed a larger frontocentral N2 and P3 ERP component for No-Go targets compared to Go targets. Previous work has shown that both components are strongly associated with the activation of inhibitory control in prefrontal cortex [33], [34], [35], [36], [37], [38], [42], [43], [44], [45]. Interestingly, a similar pattern of EEG activity was observed when comparing unconscious No-Go primes with unconscious Go primes; both components were observed to be smaller, but peaked at the expected scalp sites and at similar latencies (although the P3 peaked relatively early in the unconscious comparison). Previous imaging work using fMRI has demonstrated that unconscious response inhibition is associated with increased activation in the inferior frontal cortices (bordering anterior insula) and the pre-supplementary motor area (pre-SMA) [22].

Importantly, these EEG results could not be caused by differences in low-level stimulus characteristics between conditions or by low-level stimulus priming effects caused by prime-target congruency differences because prime-target (in)congruency and all low-level stimulus features were evenly balanced across conditions (see Table 1 and Methods). It is also very unlikely that our behavioral results were driven by feature priming. If so, we would expect a consistent pattern in the behavioral data, namely stronger priming effects for congruent prime-target pairs compared to incongruent prime-target pairs. However, inhibition rates to the target increased when the prime and the target were congruent (NoGo-NoGo>Go-NoGo), whereas we observed RT slowing effects to Go targets when the prime and the target were incongruent (NoGo-Go>Go-Go). This is in line with an inhibition account (if the prime is associated with inhibition it triggers behavioral effects irrespective of its physical similarities/differences to the target), but not with the feature priming account. Moreover, the finding of typical inhibition-related neural responses further suggests that our results are caused by inhibitory priming and not by low-level feature priming.

**Table 1:** Conditions and labels.

Trial number	Cue type	Prime type	Target type	Prime identity	Target identity
1	Square	Square	Square	No-Go	No-Go
2	Square	Square	Diamond	No-Go	Go
3	Square	Diamond	Square	Go	No-Go
4	Square	Diamond	Diamond	Go	Go
5	Diamond	Square	Square	Go	Go
6	Diamond	Square	Diamond	Go	No-Go
7	Diamond	Diamond	Square	No-Go	Go
8	Diamond	Diamond	Diamond	No-Go	No-Go

*Note*: 2×2×2 experimental design leading to eight conditions. All trials differed in the type of stimuli that were presented and resulted in different trial identities with respect to the prime and the target.

Previous research has highlighted the importance of (strong) S-R associations in the impact of unconscious stimuli on brain and behavior [24], [25], [26], [27], [46]. To illustrate, Damian (2001) investigated the role of S-R mappings during masked semantic priming and showed that priming effects disappear when the prime words were not part of the response stimulus set (see also Greenwald, 2000). Along similar lines, category set size matters [47], it has been shown that for small stimulus categories (e.g. numbers between 1 and 9) masked priming effects are typically stronger than for larger categories (e.g. animals), probably because S-R mapping can more easily be formed for smaller categories [30]. These results suggest that, in some occasions, masked unconscious primes might directly trigger specific responses while bypassing any semantic analysis [13].

However, previous evidence as well as the present results indicate that that is not all there is. For example, Klauer and colleagues (2007) [48] showed category priming for subliminal words that were never encountered consciously, when controlling for confounding factors such as word fragments and even when using words as primes and pictures as targets. Further, neuroimaging studies provided evidence for a semantic analysis of masked words [48], [49]. For example, Kiefer and colleagues have reported a series of studies in which they have shown that prime words are still processed semantically, as reflected in an enhanced N400 ERP component to incongruent prime-target pairs (e.g. “dog-chair”) compared to congruent prime-target pairs (e.g. “table-chair”) [1], [50]. A recent meta-analysis on masked priming effects [30] has nicely bridged both accounts and revealed that prime novelty indeed explains some of the variance in the reported effect sizes: strong S-R binding leads to larger effects. However, significant priming can also be observed for novel primes that are never encountered consciously.

Here, we took a somewhat different approach to study the complexity and flexibility of unconscious information processing. To do so, we mapped an arbitrary stimulus (square/diamond) to either a left- or right-hand response. Although the stimulus set-size used was small (it consisted of only two prime stimuli) and was clear right from the start, subjects had to consciously update their stimulus response mappings on every trial. In this way, S-R associations could not be established through practice. Our results suggest that unconscious information is processed in a flexible and adaptive manner. Apparently, a consciously instructed task-set can rapidly determine the processing routes taken by an unconscious stimulus [10], [31] and even when a stimulus is not consistently associated with a (No-Go) response it can reach the highest levels of the cortical and cognitive hierarchy. This paints a picture of relatively flexible and goal-directed processing of unconscious information, pushing even further the already smart characteristics revealed recently, such as top-down effects of temporal attention [1], [2], [3], spatial attention [4], [5], [6], [7], [51], task strategy and intentions [13], [14], [15], [16] on the processing of unconscious information.

We would like to note that, in the present study, the specific task-set was always instructed consciously (the cue was always conscious) and an important next step is to determine whether an unconscious stimulus can also instruct the task-set in itself or whether that process is truly restricted to conscious information [52].

## Materials and Methods

### Ethics statement

All procedures were executed in compliance with relevant laws and institutional guidelines and were approved by the ethics committee of the Psychology department of the University of Amsterdam. Subjects gave written informed consent before experimentation.

### Participants

Twenty-seven undergraduate psychology students of the University of Amsterdam (20 females, age 19–26) participated in this experiment. All were right handed, had normal or corrected-to-normal vision, and were naïve to the purpose of the experiment.

### Stimuli and procedure

White stimuli (188.4 cd/m^2^) were presented against a black background (0.44 cd/m^2^) at the center of a 17 inch DELL TFT monitor with a refresh rate of 60 Hz. The monitor was placed at a distance of ∼90 cm in front of the participant so that each centimeter subtended a visual angle of 0.64°. Participants were instructed to respond as fast as possible to a Go target by pressing a button with their right index finger and to withhold their response when they perceived a No-Go target. The target could be a diamond or a square shape (see Fig. 1a, visual angle 2.1°, duration 200 ms). Crucially, at the beginning of each trial an instruction cue (visual angle 1.78°, duration 750 ms) signaled whether the square or the diamond functioned as the No-Go target in the upcoming trial. A prime (square or diamond, visual angle 1.78°, duration 16.7 ms) was presented briefly before the target and was perfectly masked by the meta-contrast target as evidenced by chance performance on a 2-choice discrimination task administered after the main experiment (see Results). The blank interval after target presentation was jittered pseudo-randomly between 800–1400 ms (in steps of 200 ms). In sum, the paradigm constituted a 2×2×2 design resulting in eight trial types (see Table 1). Stimuli were presented using Presentation (Neurobehavioral Systems).

Data were gathered in a single EEG session (approximately two hours) in which participants performed eight experimental blocks, each containing 112 trials (80 of which contained a Go target, 32 a No-Go target). Before starting the experimental session, participants received 224 practice trials (two blocks). In 50% of the trials there was a No-Go prime presented before the target, whereas the other 50% of the trials contained a Go prime. After each block, participants received performance feedback on the targets (mean reaction time [RT] and percentage correct stops on No-Go targets). They were not informed about the presence of the primes until after the Go/No-Go task. Then, participants performed a 2-choice discrimination task to assess the visibility of the primes. This was done at the end of the Go/No-Go task to control for any learning effects of prime discrimination during task performance. Stimulus presentation and timing were exactly the same as in the Go/No-Go task. Before starting the discrimination task participants were informed about the presence of a prime appearing very shortly before the target during the Go/No-Go experiment. None of the participants reported to be aware of the primes during the Go/No-Go experiment. The 2-choice discrimination task consisted of 56 masked squares and 56 masked diamonds presented in random order. Participants were instructed to ignore the target and press the left button when they thought that a square prime was presented and press the right button when they thought a diamond prime was presented (target button assignment was counter-balanced across subjects).

### Behavioral analysis

To examine the effect of unconsciously triggered response inhibition across subjects t-tests (two tailed) were performed on mean RT on Go targets preceded by a Go versus No-Go prime. Further, it was also tested whether square root inhibition rates were higher when a No-Go target was preceded by a No-Go compared to a Go prime. Reaction times <100 and >1000 were excluded from all analyses. Detection performance (percentage correct) was tested for significance for each individual participant using a binominal test evaluated at a *p-*value of 0.05.

### EEG measurements and analyses

EEG was recorded and sampled at 1048 Hz using an ANT 64-channel system (ANT - ASA-Lab system of ASA). Sixty-four scalp electrodes were measured, as well as four electrodes for horizontal and vertical eye-movements (each referenced to their counterpart). After acquisition, EEG data was down-sampled to 256 Hz, referenced to the average of all channels and filtered using a high pass filter of 0.5 Hz, a low-pass filter of 30 Hz and a notch filter of 50 Hz. Eye movement correction was applied on the basis of Principal Component Analysis (PCA) by selecting EEG segments highly contaminated with eye blinks across recordings (spatial distribution visually inspected as being eye blinks). Principal Components Analysis method was used to determine the topographies of the artifact-free brain signals and the artifact signals. Finally, the artifact components were removed [53], [54]. Artifact correction was applied on all separate channels by removing segments outside the range of ±50 µV or with a voltage step exceeding 50 µV per sampling point. Baseline correction was applied by aligning time series to the average amplitude of the interval from 100 ms to the onset of the prime. All preprocessing steps were done using Brian Vision Analyzer (BrainProducts) and ASA (ANT-ASA-Lab).

To study the neural mechanisms of consciously triggered response inhibition we compared ERPs on trials containing a No-Go target (Table 1: trials 1, 3, 6 & 8) and trials containing a Go target (Table 1: trials 2, 4, 5 & 7). By this means we canceled out any effects caused by the primes. As common in Go/No-Go experiments, we only included inhibited No-Go trials in all EEG data analyses. To avoid a prime imbalance due to different inhibition rates caused by prime identity (see fig. 1B), we equalized the number of trials from both conditions by randomly sampling the condition containing the most correctly inhibited trials (typically No-Go targets preceded by No-Go primes) until this was equal to the condition with the smallest number of trials (typically No-Go targets preceded by Go primes).

By comparing ERPs on No-Go prime trials (Table 1: trials 1, 2, 7 & 8) with Go prime trials (Table 1: trials 2, 4, 5 & 6) we investigated the neural mechanisms of unconsciously triggered inhibition (and average out the effect of target stimuli). By collapsing across prime or target identity (depending on the performed analysis) we cancel out the contribution of any low-level differences in stimulus presentation between conditions as well as any contribution from prime-target congruency or incongruency, thus ruling out that the EEG results are due to low level stimulus priming effects. All EEG analyses were conducted on difference waves (No-Go condition minus Go condition).

Numerous experiments have investigated the neural basis of response inhibition in the Go/No-Go task and revealed the involvement of a large frontoparietal network, including middle, inferior and superior frontal cortices, the pre-supplementary motor area, the anterior cingulate cortex and several basal ganglia structures [42], [43], [44], [45]. Further, electroencephalographic (EEG) recordings showed that response inhibition on No-Go trials is typically related to two Event-Related Potential (ERP) components: a frontocentral N2 component (a negative peak around 200–300 ms after No-Go signal presentation) and a centroparietal P3 component (a positive peak around 300–500 ms after No-Go signal presentation) [33], [34], [35], [36], [37], [38]. Although the neural generators of the N2 and the P3 have not been localized with certainty, it seems likely that they originate in (or at least rely strongly on) prefrontal cortex (PFC) [33], [36], [37], [38].

To increase the signal-to-noise ratio, we created a region of interest based upon the typical spatial distribution of the N2/P3 component observed in many previous studies [33], [34], [35], [36], [37], [38] as well as visual inspection of these components in the present experiment (incorporated electrodes: Fz, F1, F2, FCz, FC1, FC2, Cz, C1 and C2). On this cluster of electrodes, we performed random-effects analyses by applying sample-by-sample paired t-tests, two-tailed around the peaks of interest (N2/P3) to test at which time points the conditions differed significantly (*p*<0.05) from zero [39]. The significant interval for the N2 and P3 was defined as all bordering significant samples around the peak of interest. All EEG analyses were performed using Matlab (Mathworks). A repeated measures ANOVA was performed using the mean activity in the significant time-window of the unconsciously initiated N2 and P3.
